# Low-Cost EEG Multi-Subject Recording Platform for the Assessment of Students’ Attention and the Estimation of Academic Performance in Secondary School

**DOI:** 10.3390/s23239361

**Published:** 2023-11-23

**Authors:** Victor Juan Fuentes-Martinez, Samuel Romero, Miguel Angel Lopez-Gordo, Jesus Minguillon, Manuel Rodríguez-Álvarez

**Affiliations:** 1 Department of Computer Engineering, Automation and Robotics, Research Centre for Information and Communication Technologies (CITIC-UGR), University of Granada, 18014 Granada, Spain; manolo@ugr.es; 2 Department of Signal Theory, Telematics and Communications, Research Centre for Information and Communication Technologies (CITIC-UGR), University of Granada, 18014 Granada, Spain; malg@ugr.es (M.A.L.-G.); minguillon@ugr.es (J.M.); 3 Neuroengineering and Computation Lab, Research Centre for Information and Communication Technologies (CITIC-UGR), University of Granada, 18014 Granada, Spain

**Keywords:** EEG, brain–computer interface (BCI), attention, academic performance, education

## Abstract

The level of student attention in class greatly affects their academic performance. Teachers typically rely on visual inspection to react to students’ attention in time, but this subjective method leads to inconsistencies across classes. Online education exacerbates the issue as students can turn off cameras and microphones to keep their own privacy. To address this, we present a novel, low-cost EEG-based platform for assessing students’ attention and estimating their academic performance. In a study involving 34 secondary school students (aged 14 to 16), participants watched an academic video and answered evaluation questions while their EEG activity was recorded using a commercial headset. The results demonstrate a significant correlation (0.53, *p*-value = 0.003) between the power spectral density (PSD) of the EEG beta band (12–30 Hz) and students’ academic performance. Additionally, there was a notable difference in PSD-beta between high and low academic performers. These findings encourage the use of PSD-beta for the immediate and objective assessment of both the student attention and the subsequent academic performance. The platform offers valuable and objective feedback to teachers, enhancing the effectiveness of both face-to-face and online teaching and learning environments.

## 1. Introduction

### 1.1. Context and Current Approaches

The attention of students during the learning–teaching process is a key factor in their academic performance. A low degree of attention usually serves as feedback or a warning sign for the instructor. This way, the teacher can switch to the most adequate teaching strategy to regain the student’s involvement in the class. Currently, the way attention is assessed in class is merely based on a visual, subjective impression. The teacher collects visual hints, such as facial expressions, gaze, or body posture. Furthermore, the growing use of online education (e-learning), lately boosted by the COVID-19 pandemic, increases the difficulty to obtain information from students about their attention level. Some recent studies show the negative consequences of e-learning during the COVID period, such as the lack of real feedback affecting the performance of the students [1] and teachers as well [2]. A very popular research topic on which many researchers have focused is the study of the relationship between performance and the level of attention of the students [3].

There are several studies on the attention span of students in class through visual observation or tests, by asking participants about their level of attention during a specific task [4,5]. Yet, this kind of subjective measure has several disadvantages [6]:

- They include systematic biases related to order, scale and halo effects, psychological factors, and others;
- These measurements are uncorrelated (and even negatively correlated) with independent, objective measures related to the variable of interest;
- These are difficult to aggregate and interpret because they are often represented in ordinal scales.

Moreover, some studies have related the academic achievement of students to emotions [7], through processing the face of the student with image recognition techniques. However, this method is a potential source of problems in the secondary school environment, as it might be against data protection laws for minors. Other studies [8] determine the state of awareness and reflections through biomarkers, such as EEG, showing that these tools can improve the teaching–learning process. EEG is a widely used tool in the clinical diagnosis of different mental diseases, like Alzheimer’s [9], attention deficit hyperactivity disorder [10], or schizophrenia [11], among other pathologies and syndromes. Furthermore, EEG has been used for the study of mental states, such as stress level [12], attention or concentration [13], and relaxation or meditation [14]. The miniaturization of electronics and the decrease in the price of the devices in the last decades have enabled the development and evolution of low-cost portable EEG (PEEG) devices, allowing their use not only in clinical diagnosis areas but as a tool for research, education, entertainment, or engineering. PEEGs are widely used for brain–computer interface (BCI) systems [15], allowing the human being to use mental states to control devices, like robots [16] or machines [17], as well as measure attention [18] or play videogames [19,20], thanks to brainwaves recorded with the EEG. The PEEG device used in this study is the Neurosky Mindwave (NeuroSky Inc., San Jose, CA, USA), which has been validated for scientific use in assessing variations in the cognitive state [21]. Some examples of research conducted with this device are: lie detection [22], the study of mental fatigue during car driving [23], or the analysis of emotions during movie viewing [24]. The Neurosky Mindwave is the cheapest PEEG device. It has a single non-invasive dry electrode and can provide objective and quantitative information to assess the level of the student’s attention in class.

Wang et al. [25] present a comprehensive and in-depth review of the state of the art regarding portable EEG devices (such as Neurosky or Emotive) in education, specifically for assessing attention. They analyzed 45 studies conducted between 2011 and 2023 that used a portable EEG for assessing attention. Four out of the total forty-five studies involved participants from secondary, middle, or high schools. Among these four studies, three of them were conducted in an online context and one of them was conducted in a naturalistic classroom (face-to-face). However, the target EEG wave was alpha. One out of the total forty-five studies used a regular classroom curriculum activity. However, the participants were university students. Fourteen out of the total forty-five studies were conducted in regular or naturalistic classrooms. Thirteen of these studies involved non-secondary school students, while one study focused on secondary school students. Once again, the target EEG wave in that study was alpha.

In conclusion, none of the experiments conducted up to this point have met the following conditions: secondary school students (14–16 years old), curricular content, class-based and assessable activities, a real classroom setting, simultaneous recording of multiple students, measurement of beta waves, and real-time information about attention levels. Only one study, conducted by Dikker et al. [26], is most similar to the present work. However, Dikker focused on analyzing students’ attention with a focus on the alpha wave. Dikker’s work aimed to analyze how attention decreases as the power of the alpha wave increases. In our research, we correlate attention with an increase in the power of beta waves.

Other studies have employed simultaneous multi-subject EEG recording techniques [27] using devices like the Neurosky headset, integrating data from all participants [28]. However, the EEG values from the participants in these studies were recorded using the manufacturer’s proprietary software, which is not publicly available or well-known, making validation impossible.

Given this, technologies such as the PEEG have the potential to help teachers to obtain a quantitative level of attention, or mental cognitive feedback from their students in real-time. Teachers can use this information accordingly. Moreover, the increasing availability of low-cost PEEG devices that are easy to deploy in a realistic classroom or e-learning setting (each student can have a PEEG device at home) makes this tool affordable for all kinds of education centers, even those with fewer resources.

### 1.2. Objectives and Expected Outcomes

The two main objectives of this work are:

(1) To develop a low-cost EEG multi-subject recording platform for the real-time assessment of students’ attention;
(2) To conduct an experiment with secondary students in a real classroom, with curricular content, as an assessable activity and record multiple subjects simultaneously, in order to validate the EEG platform as a reliable and useful tool to measure the attention and helping teachers anticipate the academic performance of their students.

The innovation of our work lies in the fact that the EEG platform was implemented for educational purposes with a low-cost non-invasive EEG device and a standard PC as the processing server. These features make this platform affordable for any educational center. As far as we know, we do not know a specific platform (hardware–software) that can be deployed in a classroom to monitor attention and can estimate the academic performance of the students on the basis of their EEG. In addition, according to our current knowledge, this is the first experiment conducted under the conditions described above. Once this technology is integrated into the classroom, a new horizon of possibilities and uses emerges, such as the study of different aspects of the teaching–learning process, such as the influence of physical activity on the attention of the student or how the class schedule affects student performance.

In this work, we intend to address the limitations of previous research, focusing on the following aspects:

- Developing a pioneer and specialized platform for this study, avoiding the use of the manufacturer’s processing application;
- Conducting experiments in a realistic environment;
- Carrying out the experimentation using assessable curriculum content;
- Conducting the experimentation with students in compulsory secondary education;
- Simultaneous recording of multiple subjects.

## 2. Materials and Methods

As mentioned in the Introduction, the main objectives of this work are to develop a low-cost EEG multi-subject recording platform for the real-time assessment of students’ attention and to conduct an experiment with secondary students in a real classroom. The purpose of the experiment was to check whether the EEG platform (developed specifically as an educational tool for teachers) can really be a useful tool for teachers to estimate the level of attention of the students. We expect our platform to show that brain activity and level of attention are correlates of students’ academic performance.

The EEG platform monitors in real time the students’ EEG using a low-cost single-channel portable and non-invasive EEG device with a dry electrode, the Neurosky Mindwave (NeuroSky Inc., San Jose, CA, USA), which has a record of success in previous studies [28,29,30]. The platform records the EEG of the students, sends the data wirelessly to a server, and shows the information to the teacher in real-time through a web interface. In this way, the teacher can know quantitatively the spectral power of the beta brainwave of each student. The signal obtained from the Neurosky Mindwave headset (NeuroSky Inc., San Jose, CA, USA) was recorded as raw EEG. The fast Fourier transform (FFT) was applied to the raw signal to compute the power spectral density (PSD) of each brainwave [31]. Specifically, we extract the PSD of beta brainwave (12–30 Hz), which is related to the state of concentration, attention, or alertness [32,33].

### 2.1. Participants

Thirty-four secondary school students participated in the study (16 females and 18 males). The inclusion criteria were: student of 3rd or 4th grade, between the ages of 14 and 16, who applied for the experiment. They all presented normal or corrected sight and reported no issues during the study. Non-regular students (students with learning difficulties) who volunteered for the experiment were excluded. They went through the experiment in a real classroom, sitting in a standard chair in front of a computer. The experiment took 4 weeks, as it extended during their regular information and communication technologies (ICT) class schedule.

### 2.2. Recordings

**Figure 1:**
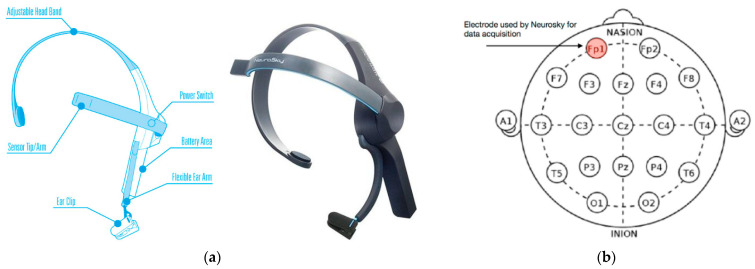
(**a**) Mindwave Mobile 2 (NeuroSky Inc., San Jose, CA, USA) and its different parts (source: Neurosky.com). (**b**) International 10–20 system for EEG recording. Inside a red circle is the FP1 position, corresponding to the pre-frontal cortex (source: bayes.acs.unt.edu).

The thirty-four EEG devices used in the experiment were Neurosky Mindwave headsets (NeuroSky Inc., San Jose, CA, USA) (Figure 1a), which has a single Fp1 dry electrode. The Fp1 position corresponds to the prefrontal cortex (Figure 1b), associated with the attention, alertness, or concentration [34,35]. The processing chip inside the Neurosky is the TGAM1 (NeuroSky Inc., San Jose, CA, USA). This module measures voltage difference between the Fp1 electrode and the metal ear clip, which acts as a ground. The raw values need to be converted to µV values. The Neurosky Support Site [36] details conversion procedure, as follows:

volts = (rawValue × (1.8/4096)/2000),(1)

This is due to a 2000 gain, 4096 value range, and 1.8 V of input voltage. The sample frequency is 512 Hz. The amplitude of waves of beta band is mostly below 30 µV [37]. The literature reports values between 50 µv and 100 µV [38] to establish a threshold for artifact rejection, such as eye blinking. In this study, we established the threshold at 75 µV.

### 2.3. Experimental Design

#### 2.3.1. Platform Architecture

**Figure 2:**
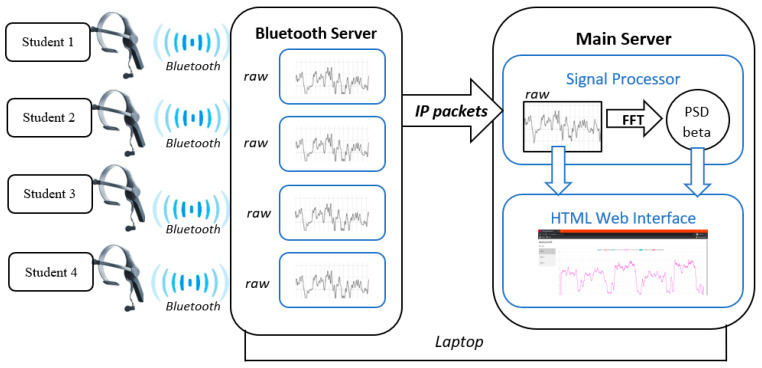
System architecture. In this scenario, four clients connect to the Bluetooth server through Bluetooth. This server sends the EEG clients data to the main server, using IP packets. Main server processes the signal, computing the PSD of each two-second epoch and produces the HTML web interface.

**Figure 3:**
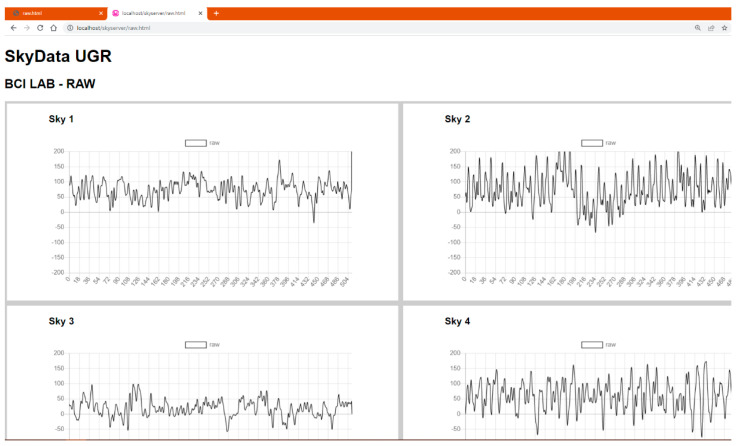
A section of the HTML web interface. It shows the raw signal, corresponding to one-second epoch of 512 values, received directly from the Neurosky (NeuroSky Inc., San Jose, CA, USA) of each participant in real time. Raw values are represented in the vertical axis (vendor units). In order to convert raw values into voltage, Formula 1 must be applied.

**Figure 4:**
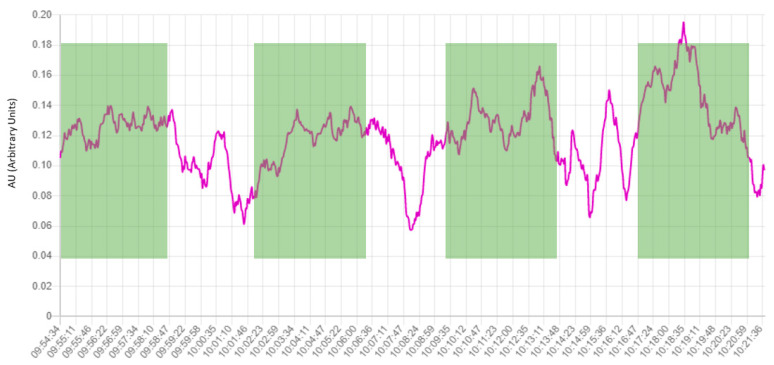
Temporal evolution of beta band PSD. In this illustrative example corresponding to one of the participants, each point represents the PSD of beta in this instant, computed from a two-second epoch. The information is shown in real-time. The green rectangles represent the time blocks when the student is visualizing the video projected over the teacher’s whiteboard (see Figure 5, green boxes). A decrease in beta PSD can be observed during the time that students are not visualizing the video.

The architecture of the platform is based on a client–server model. There are two servers: the Bluetooth server and the main server. The architecture can be deployed in two computers (one for each server) or just in one, as shown in Figure 2. Each Neurosky headset (NeuroSky Inc., San Jose, CA, USA) (client) is connected to the Bluetooth server. This server handles 4 clients, receiving the data and packaging it in IP packets that are sent to the main server. The main server has two modules: the signal processor and the HTML web interface. The signal processor module receives the packets from the Bluetooth server (raw data) and computes the PSD of the different brainwaves, in epochs of two seconds of length. After that, this information is sent to the HTML web interface to be plotted. The web interface is available in real time and shows the raw EEG signal (Figure 3) and the PSD of beta (Figure 4) along the time.

The software of the platform has three main components:

- Bluetooth server;
- Main server: signal processor;
- Main server: HTML web interface.

The Bluetooth server was programmed in Python language (3.10.11), using NumPy (1.24.3) and matplotlib.pyplot (3.7.3) libraries.

The main server has two modules. The signal processor was programmed in Python language. It uses the SciPy (1.11.1), NumPy (1.24.3), and Pingouin (0.5.3) libraries. The communication between the signal processor and the HTML web interface was made through standard text files.

The HTML web interface was programmed in JavaScript and HTML. It uses the Chart.js (2.9.3) library to plot the data and WampServer (3.0.6) to run the webpage. The HTML web interface can be opened in any browser.

The software used to screen the video on the teacher’s whiteboard and synchronize the student computers with the surveys was Edpuzzle (EDpuzzle Inc. San Francisco, CA, USA). This software allows us to insert quizzes into a chosen point of a video file.

#### 2.3.2. Experimental Procedure

The platform was tested by conducting an experiment in a realistic environment with three or four participants simultaneously. The experiment consisted of the visualization of a video with curricular content about general science of third grade, so that all students faced the task under the same conditions. In the video, there was a host that explained different Science concepts and performed some experiments. The video was projected over the main teacher’s whiteboard. The students watched the video from their seats. During the task, the students were requested to answer questions about the content. They were told that their answers would be graded, and the final score would be part of their final assessment, thus, motivating them to achieve a greater commitment.

**Figure 5:**
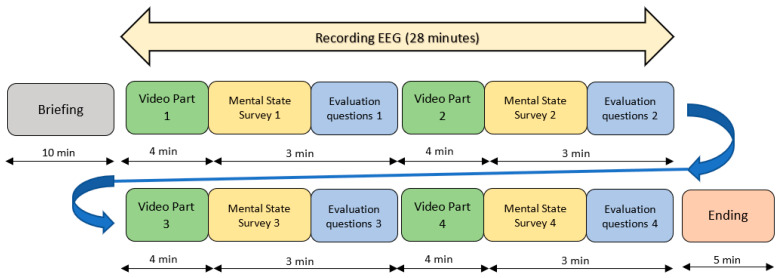
Experimental procedure. In grey, at the beginning, the briefing. The green boxes represent the visualization of the different video segments. Right after, in yellow boxes, the mental state surveys, and after them, in blue, the evaluation questions about the video content. During this time the EEG is recorded (yellow arrow above). At the end of the experiment, the students remove the headset and switch off the computers (orange box).

**Figure 6:**
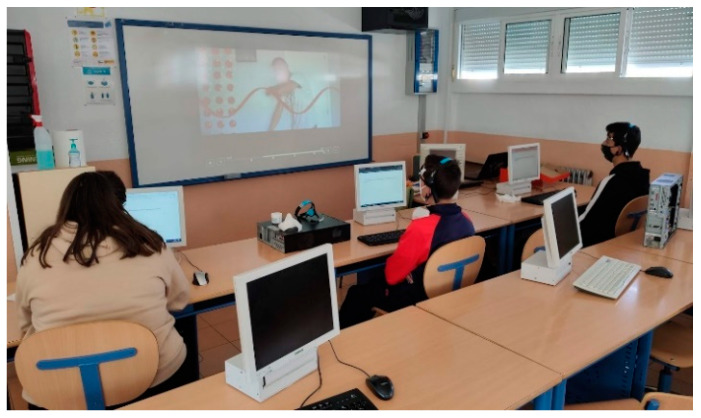
Students sitting in front of a computer watching the video on the teacher’s whiteboard, while EEG is being recorded through Neurosky headsets (NeuroSky Inc., San Jose, CA, USA).

Participants were located in front of a workstation of the information and communication technology lab (ICT-Lab) (Figure 6) and waited for the initial briefing during the first 10 min (Figure 5, grey box). During the briefing, they were informed about the experiment and the EEG headset was adjusted. The experiment did not begin until all the participants were ready.

The experiment had a duration of 28 min. The video lasted 16 min, and it was divided into 4 segments of 4 min each (Figure 5, green boxes). When a four-minute video segment ended, the student’s computer screen displayed a digital survey (Figure 5, yellow boxes). Then, they were asked to answer 6 questions (Table 1) about their emotional and mental state. All questions were related to the past four-minute video segment. Although there are many types of tests for attention level assessment, there is no universally employed test or unified method. Some studies use the NASA-TLX test [39] to measure and conducting a subjective mental workload assessment. Other studies use a simple Likert-type scale survey [40], which is a type of psychometric response scale in which responders specify their level of agreement to a statement typically in five points. We chose to use a kind of five-level Likert-type scale with simple questions because we concluded these questions were the most suitable for the students to comprehend.

Once the students completed the mental state questions, they had to answer 4 more questions about the contents of the video segment that was just watched (Figure 5, blue boxes). These were multiple-choice questions with penalization for wrong answers, to avoid the randomness in the answers. In total, they had 3 min to answer all questions (6 of mental state and 4 of evaluation). The video was synchronized with the surveys. Right when a four-minute video segment was finished, the surveys appeared on the screen of the students’ computers, automatically followed by the evaluation questions. Once the evaluation of those questions concerning the last four-minute video segment ended, the next four-minute video segment started. The process was repeated for the remaining 3 segments. Although there were 28 min of EEG recorded, only the 16 min concerning the video segments were considered for the experiment (Figure 5, green boxes). When the experiment had concluded, the students took off their EEG headset, switched off the computer, and went back to their class (Figure 5, orange box).

In addition to the literature supporting the relation between beta and attention, the real-time EEG platform also shows changes in the attention during the experiment. For example, it can be observed how the PSD of beta increases during the temporary blocks in which the students are viewing the video, and decreases when they begin to answer the questions (Figure 4).

### 2.4. Signal Processing and Statistical Analysis

The aim of signal processing is to obtain the PSD of the beta band (12–30 Hz) for each two-second epoch, as previously performed in other studies [41]. In addition, the mean value of the answers for each mental state question (Table 1) was calculated. These 6 final values were represented by a decimal number between 1 and 5.

The following method was applied to compute the PSD of beta band for a 2 s epoch: filtered by means of a Butterworth bandpass filter between 3 Hz and 40 Hz, detrend filtered and z-scored (it removes the mean and normalized by the standard deviation), Tukey window (fraction 0.15), and the fast Fourier transform (FFT) based on Welch’s method (50% overlap). At the end of the experiment, the sum of all PSD values (1 of each 2 s epoch) is calculated as the total mean PSD of beta for each student. The total mean PSD of beta value was normalized between 0 and 1, and it represents a rough estimation of the average level of attention of the student during the task.

Since the purpose of this tool is for both face-to-face and online classes, we have taken into account beta as the only EEG marker for the assessment of attention, discarding visual inspections (only available in face-to-face lessons).

**Figure 7:**
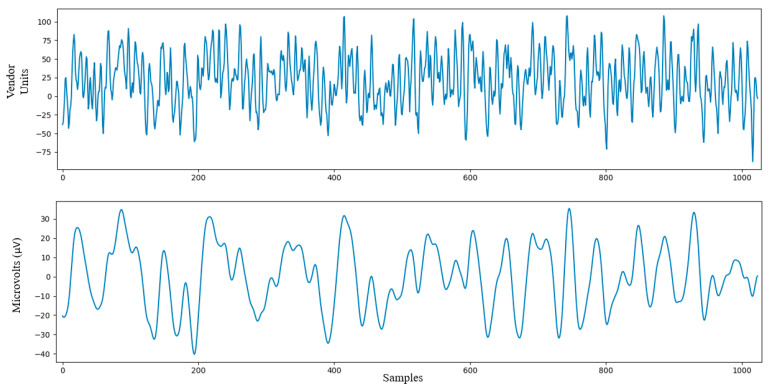
Signal processing. As an illustrative example of one epoch of two seconds with 1024 samples. The upper and bottom plots show the raw and processed (after noise removal) EEG signals, respectively.

In addition, due to the fact that the objective of the experiment was to test the tool in a realistic classroom, the EEG signal was exposed to natural noise on purpose. Thus, the conditions of the experiment were hardened, making the platform more robust. With this in mind, and assuming that the level of noise in EEG signals could not be known or controlled a priori, our strategy could never succeed by direct noise confrontation. Instead, we decided to implement a conservative and robust methodology able to coexist with relevant levels of noise, although at the cost of suboptimal performance. In this sense, we decided to implement several techniques: bandpass filter 3–40 Hz (this band eliminates both 50 Hz from the power grid and flicker, typically centered one octave lower); elimination of epoch above 75 µV, which also eliminated very energetic impulsive sources of short duration and wide spectrum with beta contribution (such us eye blinking); averaging of 16 min of the PSD of each two-second epoch; and Z-score of each epoch, which ensures that highly energetic and localized impulsive noise sources, such as those that occur with experimentation outside the laboratory, do not have a greater contribution than any other epoch. Figure 7 shows raw signals corresponding to two-second epoch (1024 values) before and after the signal processing and noise filtering.

The total mean PSD of beta for each student was correlated with the task score and the mean of the answers to each mental state question, using the following correlation models: Pearson (linear), Spearman (non-linear), and skipped. A skipped correlation is a robust generalization of Pearson’s r or Spearman’s r by measuring the strength of the linear association, ignoring outliers detected by taking into account the overall structure of the data [42,43].

Next, in Table 2, all the signal processing specifications are summarized.

**Figure 8:**
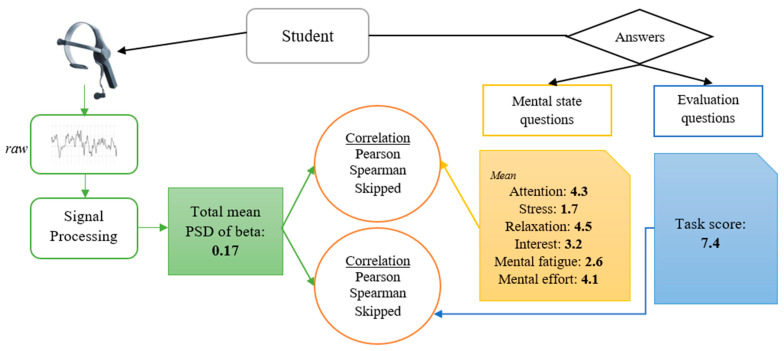
Data obtained after the signal processing. In green, the total mean PSD of beta. In yellow, the mean of the answers to each mental state question (1–5). In blue, the task score obtained from the evaluation questions (0–10).

Figure 8 shows an example of the different values obtained at the end of the signal processing.

In addition to the continuous analysis based on different types of correlation, a statistical group analysis was performed in order to elucidate if there was statistically significant differences in the total mean PSD of beta between the worst and the best performers (i.e., lowest and highest task scores, respectively). Only students with task scores out the range mean ±0.5 were included in this analysis. That is, students with scores close to the mean score were excluded since they cannot be considered as worst or best performers. Moreover, students with the total mean PSD of beta outside of the [5–95]% range were considered outliers and removed (2 of each group). After checking that the parametric requirements of normality (Shapiro–Wilk test) and variance homogeneity (Bartlett test) of both distributions were not met, a nonparametric statistical test was performed. In particular, the Mann–Whitney U-test was used to test the null hypothesis that the total mean PSD from groups are samples from continuous distributions with equal medians. We set the significance level at 5% (i.e., *p*-value < 0.05).

Alternatively to correlation and statistical group analysis, we defined a classification model based on a neural network (multilayer perceptron) [44] to discriminate students that passed or failed the test (19 and 15 students passed and failed, respectively). The optimization of the neural network model involved a thorough exploration of hyperparameters through a grid-search technique. To validate the model, we utilized a leave-one-out method, conducting 34 iterations: in each iteration, one case served as the test set, while the remaining 33 formed the training set. Subsequently, using the collective predictions from these iterations, we constructed a receiver operating characteristic (ROC) curve, and computed the area under the curve (AUC). Furthermore, we employed a confusion matrix to ascertain recall, precision, accuracy, and F1 score.

## 3. Results

### 3.1. Continuous Analysis

Table 3 shows the correlation between the total mean PSD of beta (Figure 8, green box) and the other values obtained from student’s answers: task score (Figure 8, blue box) and subjective mental state questions (Figure 8, orange box). The three models used for correlation were Pearson, Spearman, and skipped.

The best model to correlate the PSD of beta with the task score was skipped, with a correlation of 0.53, a CI95% between 0.2 and 0.75, and a *p*-value of 0.003. Skipped detected and removed 5 outliers from the data set, obtaining better results than Pearson or Spearman. The correlation between the total mean PSD of beta and any of the mental state questions is less than 0.22, regardless of the correlation model. The *p*-value in all cases is higher than 0.21.

Table 4 shows statistical information about the different data.

**Figure 9:**
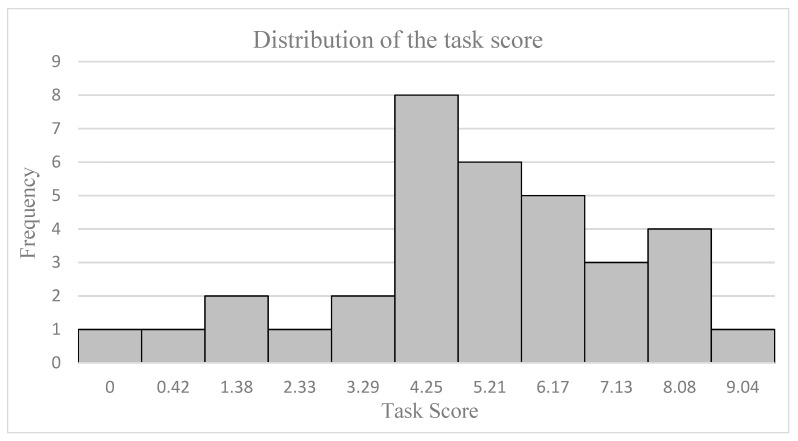
Distribution of the task score. The *x*-axis represents the students’ scores and the *y*-axis represents the frequency of these scores. The Gaussian-shaped distribution of scores suggests that the difficulty and the content of the task are coherent with regular experience in a typical class.

Figure 9 shows the distribution of the task score. A total of 55% of the students passed the examen. The most repeated punctuation was 4.25.

**Figure 10:**
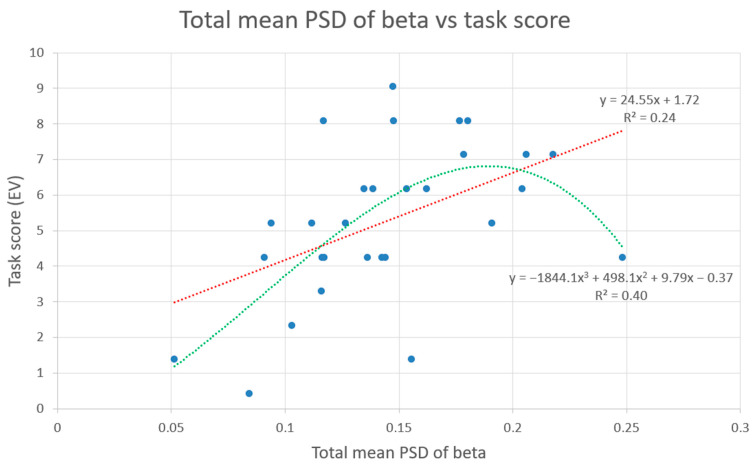
Scatter plot of the total mean PSD of beta and the task score of the participants after removing the outliers. The *x*-axis represents the beta levels, and the *y*-axis represents the score of the evaluation test. Two trend lines have been plotted, a linear (red) and a non-linear (green). The polynomic regression has a better fit than the linear one (R^2^ = 0.42).

Figure 10 shows the scatter plot of the total mean PSD of beta band and the task score for each student. Two trend lines have been plotted: a linear regression (red) a polynomic regression (green). The polynomic regression (R^2^ = 0.42) has a better fit than the linear one (R^2^ = 0.24).

### 3.2. Group Analysis

**Figure 11:**
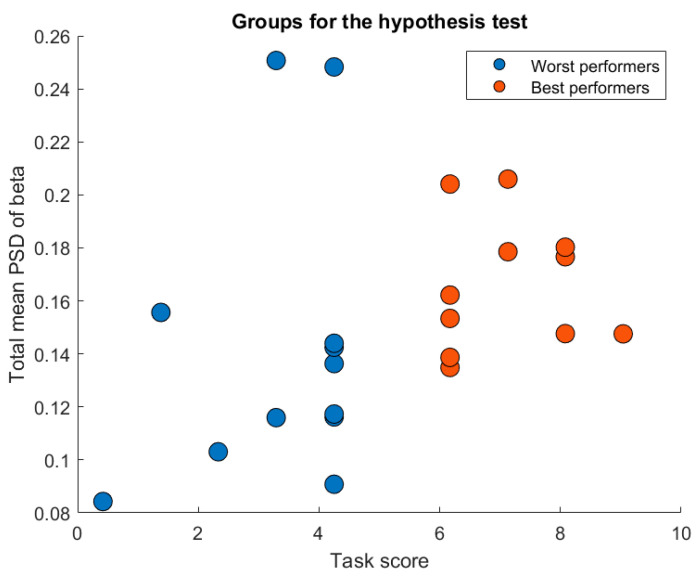
Groups conformation for the statistical group analysis. In blue, worst performers (scores under 5). In red, best performers (scores above 5). After removing the outliers, it is generally higher the beta levels of the best performers than the worst performers.

Figure 11 shows the groups conformation for the statistical group analysis. The final sizes of the groups were 12 and 11 for the worst and best performers, respectively.

**Figure 12:**
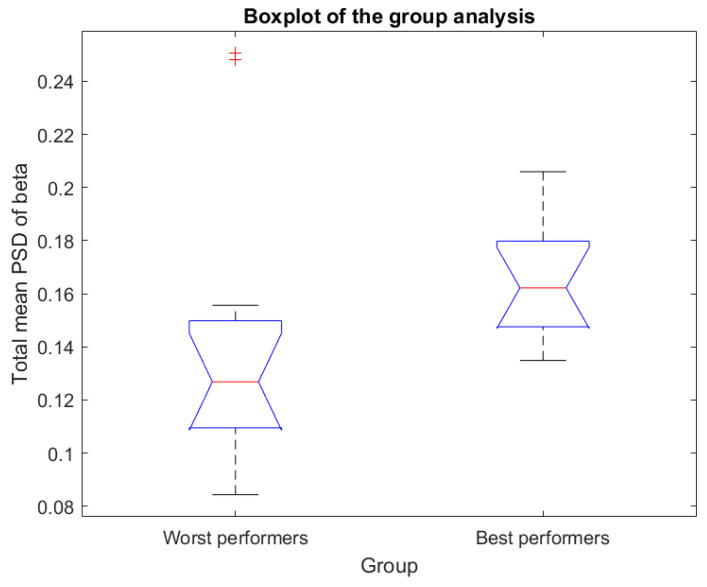
Boxplot of the group analysis. + indicates data points beyond the whiskers (maximum whisker length is equal to the interquartile range). The median of the best performers is approximately 0.16, while the median of the worst performers is approximately 0.12.

Figure 12 shows a boxplot of the group analysis. The Mann–Whitney U-test rejected the null hypothesis that the total mean PSD of beta from groups are samples from continuous distributions with equal medians with a *p*-value < 0.05. Therefore, the medians of worst and best performers are different with statistical significance.

### 3.3. Classification Model

Table 5 compiles the outcomes derived from the confusion matrix and the values of evaluation metrics, including recall, accuracy, precision, and F1 score.

**Figure 13:**
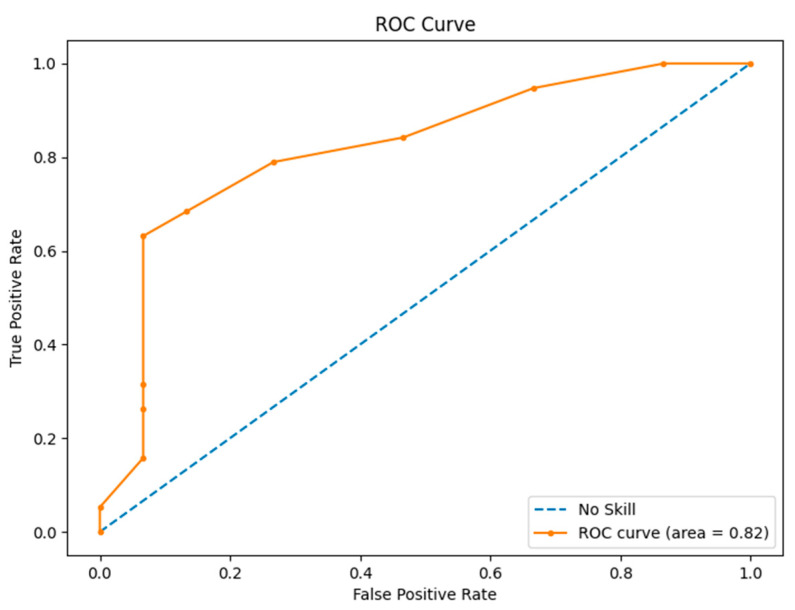
ROC curve and AUC.

Figure 13 displays the ROC curve alongside the area under the curve (AUC).

## 4. Discussion

In this study, an EEG-based platform was used under realistic conditions to extract the levels of attention of a class and to relate them to the academic performance of students. These results evidence that our platform is a feasible tool for the estimation of the students’ academic performance during a class, and the consequential potential to improve the effectiveness of the teaching–learning process and, hence, the academic outcomes.

Additionally, given the assumption that the level of noise in EEG signals may not be predetermined or managed beforehand, we have adopted a conservative and robust methodology employing multiple techniques. This approach allowed for coexistence with substantial noise levels, albeit at the expense of optimal performance. Consequently, we have substantiated our primary assertion: the development of a platform capable of functioning in conditions akin to those in a real secondary class environment. This platform exhibits a reasonable degree of resilience and adaptability, even in the presence of expectedly high levels of EEG noise.

The results (Table 3) show a significant correlation in the skipped model between the students’ task score and the total mean PSD of beta (R = 0.53, *p*-value = 0.003). As expected, the skipped model obtained better results than Pearson or Spearman model, since this method is more robust, and has the capacity to detect and remove outliers. A positive correlation between the total mean PSD of beta band and the task score across students indicates that students with higher PSD of beta will achieve better academic results. Under the assumption that students with higher levels of attention will obtain better results [4], our finding is coherent with the literature, which relates enhanced levels of beta with those of concentration, alertness, and attention [33,34,35,36].

Table 3 also shows the correlation between the mean values of the answers to the subjective questions about the mental state (see Table 1) across students. For them, no significant correlation was found with the PSD of beta (the null hypothesis was zero correlation). Furthermore, none of the correlations have statistical significance, as all the *p*-values of the correlations are greater than 0.05. Since the significant correlation between PSD of beta and academic performance was established in this study, the former suggests that the students’ subjective perception cannot be considered as a good indicator of attention. In other words, asking students about their mental state (e.g., level of attention, interest, mental fatigue, etc.) is not helpful to estimate academic performance at the end of a class.

In Table 4 and Figure 9, the mean task score (5.0, s.d. 2.3), the Gaussian-shaped distribution of scores centered at the mean task score, and the high values achieved by related items (interest = 4.2, attention = 4.3) suggest that the difficulty and the content of the task were coherent with regular experience in a typical class. In addition, the students did not experience high mental fatigue or effort (2.1 and 2.6, respectively) and the level of stress and relaxation were low and high, respectively (1.6 and 3.2, respectively). These results reveal that the study was conducted in adequate condition for the assessment of the academic performance.

Figure 10 shows two regression equations between the total mean PSD of beta and the task score: a polynomial and a linear. The polynomial regression (R^2^ = 0.42) has a better fit than the linear (R^2^ = 0.24). This aligns with the correlation values found using the skipped model, as, for this experiment, ‘skipped’ has been configured based on the Spearman model, which is non-linear.

Figure 11 shows the best scores in red (scores above 5) and the worst scores in blue points (scores below 5). Visually, it can be observed that, on average, students with higher scores have a higher total mean PSD of beta compared to students with lower scores. Indeed, Figure 12 shows that the worst performers median of the total mean PSD of beta is around 0.12 and the best performers median of the total mean PSD of beta is around 0.16.

The results of hypothesis test in the worst vs. best performers analysis demonstrate that, from a statistical point of view, the best performers group has a significantly higher total mean PSD of beta than the worst performers group. This result is consistent with the positive correlation shown in Table 3 and with the main claim of our work, since this platform can help teachers to find the students that will have better academic performance and who will not.

Table 5 and Figure 13 present the outcomes of the neural network classification model. The ROC area (0.82), precision (0.88), and F1 score (0.80) collectively indicate the platform’s capability to estimate, in real time, the likelihood of passing or failing a test centered on curricular content during a class. This is a promising result that credits our platform with the ability to yield a reasonable a priori estimation of the rate of students in the class that will pass the test, thus, constituting a valuable piece of information for teachers.

The revision of the state of art about portable EEG technology in educational research [45] points out that most of the EEG-based studies were conducted with university students, in non-naturalistic classrooms, and as an after-school activity. Just a few of them were made with curricular content. Conversely, our study was conducted with students in a secondary school, in a realistic classroom, recording 3–4 participants simultaneously, with curricular contents, during class hours, and as an assessable task. To the best of our knowledge, this is the first experiment performed under these conditions. Therefore, we cannot compare our results to similar studies, as the experimental conditions and the study objective (beta-performance) are not the same. Another relevant aspect is that the EEG platform was implemented with a low-cost non-invasive EEG device and a standard PC as the processing server. These features make this platform affordable for any educational center.

## 5. Conclusions

In this work, we have presented a new low-cost EEG simultaneous recording platform that can monitor the PSD level of beta of four students in real time while they are performing an academic task. After testing the platform, the results point out that there exists a direct relation between the beta band and the academic performance that, conveniently interpreted by teachers, can be used to steer the course of the class. Furthermore, we have demonstrated that despite being a low-cost EEG platform, the platform is functional and can help estimate students’ academic performance. As a tool, we expect an impact on the community of secondary school teachers.

This tool could be adopted in fields such as neuromarketing or in any task requiring the monitoring of real-time attention levels, such as industrial robotics, the defense sector, or the aerospace industry, where attention levels can suddenly decrease due to fatigue or stress, potentially leading to serious outcomes.

### 5.1. Limitations

This exploratory and feasibility study has some limitations, such as:

a. The number of participants that can be registered simultaneously due to the Bluetooth connection is limited to four;
b. The use of a single dry electrode, while appropriate in terms of usability and cost, limits the richness and quality of the EEG signal. Another study using more sophisticated devices, with a greater number of wet electrodes, would be possible, but it would be outside the scope of this study’s purpose, mainly due to higher costs and more difficult preparation for the user;
c. The number of participants. Although the sample size is low for solid conclusions, the results shown in this work support the relationship between the total mean PSD of beta and the academic performance;
d. The main claim of our work is to introduce a new low-cost EEG platform capable of recording, processing, and delivering valuable real-time information to teachers in a real classroom setting. Our primary focus is not on developing an optimal EEG signal processing algorithm, but to show the benefits of the use of such a platform. The efficiency of our platform could possibly improve with a more advanced technique like artificial intelligence (AI).

### 5.2. Future Work

For future studies, some improvements could be applied, such as: replacing the Bluetooth connection with WiFi, which would allow for registering a much larger number of participants simultaneously (more than 100); applying stronger statistical analysis models or AI models, such as neural networks, random forest, or support vector machines (SVM); and conducting experiments under same conditions with a larger sample to deepen the generated knowledge.

## Figures and Tables

**Table 1:** Mental state questions made to students when finishing each four-minute video segment.

Number	Question
1	What was your level of attention?
2	What was your level of stress?
3	What was your level of relaxation?
4	What was your level of interest?
5	What was your level of mental fatigue?
6	What was your level of mental effort?

**Table 2:** Specifications of signal processing.

Specification	Value
Sampling frequency per second	512
Window size (epoch)	2 s (1024 samples)
Artifact filtering threshold	75 µV
Signal discretization method	Fast Fourier transform
Signal amplitude range	−100, 100 µV
Target frequency band	Beta (12–30 Hz)
Noise filtering	Butterworth, detrend, z-scored, and Tukey window

**Table 3:** Correlation between the total mean PSD of beta and the score obtained from student’s answer: evaluation questions (task score) and mental state questions. The correlation between the task score and the total mean PSD of beta is 0.53, with a *p*-value of 0.003 (first row). There is no significant correlation between the score obtained from the mental state questions and the total mean PSD of beta (row 2 to 7).

	Model	Outliers	R	CI95%	*p*-Value	Power
Task score ^1^	Pearson	0	0.23	[−0.12, 0.54]	0.18	0.26
	Spearman	0	0.32	[−0.02, 0.6]	0.06	0.46
	**Skipped**	**5**	**0.53**	**[0.21, 0.75]**	**0.003**	**0.87**
Interest ^2^	Pearson	0	−0.04	[−0.38, 0.31]	0.81	0.05
	Spearman	0	−0.03	[−0.37, 0.32]	0.86	0.05
	Skipped	0	−0.03	[−0.37, 0.32]	0.86	0.05
Attention ^2^	Pearson	0	−0.05	[−0.39, 0.29]	0.76	0.06
	Spearman	0	−0.06	[−0.39, 0.29]	0.74	0.06
	Skipped	1	0.0	[−0.35, 0.35]	0.99	0.05
Mental fatigue ^2^	Pearson	0	0.04	[−0.30, 0.38]	0.80	0.05
	Spearman	0	0.01	[−0.33, 0.35]	0.94	0.05
	Skipped	0	0.01	[−0.33, 0.35]	0.95	0.05
Mental effort ^2^	Pearson	0	0.04	[−0.30, 0.38]	0.80	0.05
	Spearman	0	0.06	[−0.4, 0.29]	0.73	0.06
	Skipped	0	0.06	[−0.4, 0.29]	0.73	0.06
Stress ^2^	Pearson	0	−0.21	[−0.51, 0.15]	0.24	0.21
	Spearman	0	−0.22	[−0.53, 0.13]	0.21	0.24
	Skipped	0	−0.22	[−0.53, 0.13]	0.21	0.24
Relaxation ^2^	Pearson	0	0.09	[−0.26, 0.43]	0.59	0.08
	Spearman	0	−0.02	[−0.37, 0.32]	0.89	0.05
	Skipped	0	−0.22	[−0.37, 0.32]	0.89	0.05

^1^ Represented as a value between 0 and 10. ^2^ Represented as a value (between 1 and 5) obtained from the subjective mental state questions asked to the student during the experiment (Table 1).

**Table 4:** Statistical information (mean, standard deviation and median) about the data of the experiment: task score and mental state questions. The mean task score (5.0, s.d. 2.3) suggests that the difficulty and the content of the task are coherent with regular experience in a typical class.

Data	Mean	Standard Dev.	Median
Task score	5.0	2.3	5.2
Interest	4.3	0.5	4.2
Attention	4.3	0.5	4.4
Mental fatigue	2.2	0.7	2.0
Mental effort	2.7	0.9	2.6
Stress	1.7	0.6	1.5
Relaxation	3.3	0.8	3.2

**Table 5:** Confusion matrix results and values of evaluation metrics.

True Positives	False Positives	False Negatives	True Negatives	Recall	Accuracy	Precision	F1-Score
14	2	5	13	0.73	0.79	0.86	0.80

## Data Availability

The data that support the findings of this study are available on request from the corresponding author. The data are not publicly available due to privacy or ethical restrictions.

## References

[1] Feng X., Ioan N., Li Y. (2021). Comparison of the Effect of Online Teaching during COVID-19 and Pre-Pandemic Traditional Teaching in Compulsory Education. J. Educ. Res..

[2] Kulikowski K., Przytuła S., Sułkowski Ł. (2022). E-learning? Never Again! On the Unintended Consequences of COVID-19 Forced E-learning on Academic Teacher Motivational Job Characteristics. High. Educ. Q..

[3] Darvishi A., Khosravi H., Sadiq S., Weber B. (2021). Neurophysiological Measurements in Higher Education: A Systematic Literature Review. Int. J. Artif. Intell. Educ..

[4] Greene B.A. (2015). Measuring Cognitive Engagement with Self-Report Scales: Reflections From Over 20 Years of Research. Educ. Psychol..

[5] Shah S.M.H., Saleem S. (2015). Level of Attention of Secondary School Students and Its Relationship with Their Academic Achievement. J. Arts Humanit..

[6] Jahedi S., Méndez F. (2014). On the Advantages and Disadvantages of Subjective Measures. J. Econ. Behav. Organ..

[7] Bulut Özek M. (2018). The Effects of Merging Student Emotion Recognition with Learning Management Systems on Learners’ Motivation and Academic Achievements. Comput. Appl. Eng. Educ..

[8] Durall E., Leinonen T., Kravcik A.M.M., Pammer M.P.V. (2015). Feeler: Supporting Awareness and Reflection about Learning through EEG Data. Proceedings of the 5th Workshop on Awareness and Reflection in Technology Enhanced Learning.

[9] Perez-Valero E., Morillas C., Lopez-Gordo M.A., Carrera-Muñoz I., López-Alcalde S., Vílchez-Carrillo R.M. (2022). An Automated Approach for the Detection of Alzheimer’s Disease from Resting State Electroencephalography. Front. Neuroinform..

[10] Freismuth D., TaheriNejad N. (2022). On the Treatment and Diagnosis of Attention Deficit Hyperactivity Disorder with EEG Assistance. Electronics.

[11] Alves C.L., Pineda A.M., Roster K., Thielemann C., Rodrigues F.A. (2022). EEG Functional Connectivity and Deep Learning for Automatic Diagnosis of Brain Disorders: Alzheimer’s Disease and Schizophrenia. J. Phys. Complex..

[12] Minguillon J., Perez E., Lopez-Gordo M., Pelayo F., Sanchez-Carrion M. (2018). Portable System for Real-Time Detection of Stress Level. Sensors.

[13] Liu N.-H., Chiang C.-Y., Chu H.-C. (2013). Recognizing the Degree of Human Attention Using EEG Signals from Mobile Sensors. Sensors.

[14] You S.D. (2021). Classification of Relaxation and Concentration Mental States with EEG. Information.

[15] Ron-Angevin R., Lopez M.A., Pelayo F., Cabestany J., Sandoval F., Prieto A., Corchado J.M. (2009). The Training Issue in Brain-Computer Interface: A Multi-Disciplinary Field. Bio-Inspired Systems: Computational and Ambient Intelligence.

[16] Li Y., Zheng T., Wang M., Dong L., Wang P., Qin X. (2021). A Coloring and Timing Brain-Computer Interface for the Nursing Bed Robot. Comput. Electr. Eng..

[17] Sharma H., Mahajan R., Sakthivel G., Saravanakumar D., Raghukiran N. (2021). Brain Computer Interface Controlled Wheel Chair. J. Phys. Conf. Ser..

[18] Lopez-Gordo M.A., Pelayo F., Fernandez E., Padilla P. (2015). Phase-Shift Keying of EEG Signals: Application to Detect Attention in Multitalker Scenarios. Signal Process..

[19] Perez-Valero E., Lopez-Gordo M.A., Vaquero-Blasco M.A. (2021). An Attention-driven Videogame Based on Steady-State Motion Visual Evoked Potentials. Expert Syst..

[20] Amin M., Tubaishat A., Al-Obeidat F., Shah B., Karamat M. (2022). Leveraging Brain–Computer Interface for Implementation of a Bio-Sensor Controlled Game for Attention Deficit People. Comput. Electr. Eng..

[21] Rieiro H., Diaz-Piedra C., Morales J.M., Catena A., Romero S., Roca-Gonzalez J., Fuentes L.J., Di Stasi L.L. (2019). Validation of Electroencephalographic Recordings Obtained with a Consumer-Grade, Single Dry Electrode, Low-Cost Device: A Comparative Study. Sensors.

[22] Mallikarjun H.M., Manimegalai P., Suresh H.N. (2017). Machine Learning Based Classifier for Falsehood Detection. IOP Conf. Ser. Mater. Sci. Eng..

[23] Morales J.M., Díaz-Piedra C., Rieiro H., Roca-González J., Romero S., Catena A., Fuentes L.J., Di Stasi L.L. (2017). Monitoring Driver Fatigue Using a Single-Channel Electroencephalographic Device: A Validation Study by Gaze-Based, Driving Performance, and Subjective Data. Accid. Anal. Prev..

[24] KB S.K., Krishna G., Bhalaji N., Chithra S. (2019). BCI Cinematics—A Pre-Release Analyser for Movies Using H_2_O Deep Learning Platform. Comput. Electr. Eng..

[25] Wang J.-W., Zhang D.-W., Johnstone S.J. (2023). Portable EEG for Assessing Attention in Educational Settings: A Scoping Review. arXiv.

[26] Dikker S., Haegens S., Bevilacqua D., Davidesco I., Wan L., Kaggen L., McClintock J., Chaloner K., Ding M., West T. (2020). Morning Brain: Real-World Neural Evidence That High School Class Times Matter. Soc. Cogn. Affect. Neurosci..

[27] Babiloni F., Astolfi L. (2014). Social Neuroscience and Hyperscanning Techniques: Past, Present and Future. Neurosci. Biobehav. Rev..

[28] Sezer A., İnel Y., Seçkin A.Ç., Uluçınar U. An Investigation of University Students’ Attention Levels in Real Classroom Settings with NeuroSky’s MindWave Mobile (EEG) Device. Proceedings of the International Educational Technology Conference–IETC 2015.

[29] Lancheros-Cuesta D.J., Carrillo-Ramos A., Lancheros-Cuesta M. (2019). Evaluation of Content Adaptation: Case Study with NeuroSky MindWave in Children with Learning Difficulties. Int. J. Web Inf. Syst..

[30] Rebolledo-Mendez G., Dunwell I., Martínez-Mirón E.A., Vargas-Cerdán M.D., de Freitas S., Liarokapis F., García-Gaona A.R., Jacko J.A. (2009). Assessing NeuroSky’s Usability to Detect Attention Levels in an Assessment Exercise. Human-Computer Interaction. New Trends.

[31] Salabun W. (2014). Processing and Spectral Analysis of the Raw EEG Signal from the MindWave. Prz. Elektrotechniczny.

[32] Kang J.-S., Ojha A., Lee M., Arik S., Huang T., Lai W.K., Liu Q. (2015). Concentration Monitoring with High Accuracy but Low Cost EEG Device. Neural Information Processing.

[33] Wolska A., Sawicki D., Kołodziej M., Wisełka M., Nowak K. (2019). Which EEG Electrodes Should Be Considered for Alertness Assessment?. Proceedings of the 3rd International Conference on Computer-Human Interaction Research and Applications.

[34] Ulker B., Tabakcioglu M.B., Cizmeci H., Ayberkin D. (2017). Relations of Attention and Meditation Level with Learning in Engineering Education. Proceedings of the 2017 9th International Conference on Electronics, Computers and Artificial Intelligence (ECAI).

[35] Sethi C., Dabas H., Dua C., Dalawat M., Sethia D. (2018). EEG-Based Attention Feedback to Improve Focus in E-Learning. Proceedings of the 2018 2nd International Conference on Computer Science and Artificial Intelligence.

[36] NeuroSky Suppor Site How to Convert Raw Values to Voltage?. http://support.neurosky.com/kb/science/how-to-convert-raw-values-to-voltage.

[37] Kane N., Acharya J., Beniczky S., Caboclo L., Finnigan S., Kaplan P.W., Shibasaki H., Pressler R., van Putten M.J.A.M. (2017). A Revised Glossary of Terms Most Commonly Used by Clinical Electroencephalographers and Updated Proposal for the Report Format of the EEG Findings. Revision 2017. Clin. Neurophysiol. Pract..

[38] Mennes M., Wouters H., Vanrumste B., Lagae L., Stiers P. (2010). Validation of ICA as a Tool to Remove Eye Movement Artifacts from EEG/ERP. Psychophysiology.

[39] Dan A., Reiner M. (2018). Reduced Mental Load in Learning a Motor Visual Task with Virtual 3D Method. J. Comput. Assist. Learn..

[40] Edwards A.A., Massicci A., Sridharan S., Geigel J., Wang L., Bailey R., Alm C.O. (2017). Sensor-Based Methodological Observations for Studying Online Learning. Proceedings of the 2017 ACM Workshop on Intelligent Interfaces for Ubiquitous and Smart Learning.

[41] Gudmundsson S., Runarsson T.P., Sigurdsson S., Eiriksdottir G., Johnsen K. (2007). Reliability of Quantitative EEG Features. Clin. Neurophysiol..

[42] Pernet C.R., Wilcox R., Rousselet G.A. (2013). Robust Correlation Analyses: False Positive and Power Validation Using a New Open Source Matlab Toolbox. Front. Psychol..

[43] Rousselet G.A., Pernet C.R. (2012). Improving Standards in Brain-Behavior Correlation Analyses. Front. Hum. Neurosci..

[44] Taud H., Mas J.F., Camacho Olmedo M.T., Paegelow M., Mas J.-F., Escobar F. (2018). Multilayer Perceptron (MLP). Geomatic Approaches for Modeling Land Change Scenarios.

[45] Xu J., Zhong B. (2018). Review on Portable EEG Technology in Educational Research. Comput. Hum. Behav..

